# A Rare and Severe Cause of Abdominal Pain in a Hemodialysis Patient

**DOI:** 10.7759/cureus.30800

**Published:** 2022-10-28

**Authors:** Teresa Furtado, Patrícia Domingues, Ana Piedade, Lucia Parreira, Ana Natário

**Affiliations:** 1 Nephrology, Setubal Hospital Center, Setubal, PRT

**Keywords:** non-occlusive mesenteric ischemia, abdominal pain, hemodialysis, aeroportia, pneumatosis intestinalis

## Abstract

Pneumatosis intestinalis (PI) and aeroportia have been rarely described in hemodialysis patients. We present a case of a 64-year-old woman on regular hemodialysis who presented with abdominal pain, vomiting, and diarrhea. Abdominal CT showed pneumatosis intestinalis and aeroportia suggestive of ischemic abnormalities. In this case, given the absence of transmural necrosis or bowel perforation, aeroportia seemed to be caused by nonocclusive mesenteric ischemia (NOMI), an increasingly recognized complication in hemodialysis patients. The patient was proposed for emergent exploratory laparotomy; however, she had a fatal outcome. Hemodialysis-dependent patients should be considered at risk of the “low-flow syndrome” of mesenteric arterial circulation. Prevention is crucial, and early detection of these entities is important for prompt diagnosis and management of mesenteric ischemia.

## Introduction

Pneumatosis intestinalis (PI) and aeroportia are rare radiologic features that refer to the presence of gas within the intestinal wall and in portomesenteric vessels, respectively. Both are typical findings of mesenteric ischemia, the leading cause accounting for 70% of the cases [1]. Other conditions such as intra-abdominal abscesses, ulcerative colitis, small bowel obstruction, gastric ulcer, diverticulitis, and pancreatitis and following invasive procedures have also been found to be associated [2-4]. PI, which is often asymptomatic, might have a benign course depending on the underlying disease. However, when combined with aeroportia, it is strongly associated with bowel infarction, and immediate surgical intervention is required [1-2]. A contrast-enhanced CT scan is useful to establish the diagnosis, determine the underlying etiology, and identify potential associated complications. In the face of isolated PI, without signs suggestive of acute abdomen, conservative treatment can be an option. However, in the presence of other conditions, an emergent exploratory laparotomy may be required. Life-threatening PI can be suspected when confronted with bowel wall thickening, arterial or venous occlusion, soft-tissue stranding, and hepatic venous gas on a CT scan [2].

## Case presentation

**Figure 1:**
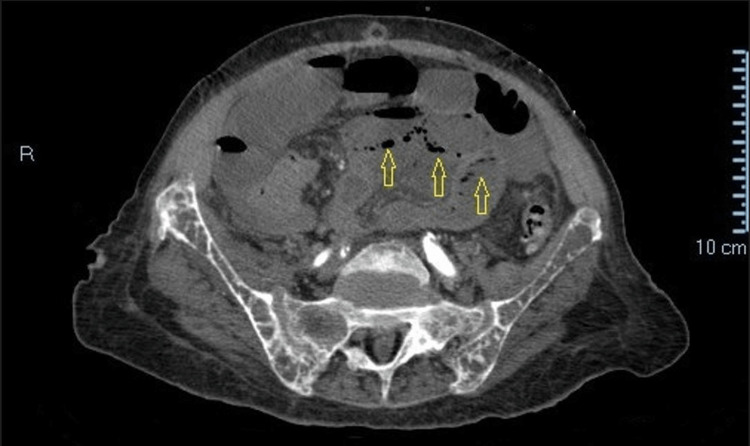
A contrast-enhanced CT scan shows diffuse intestinal pneumatosis (yellow arrows)

**Figure 2:**
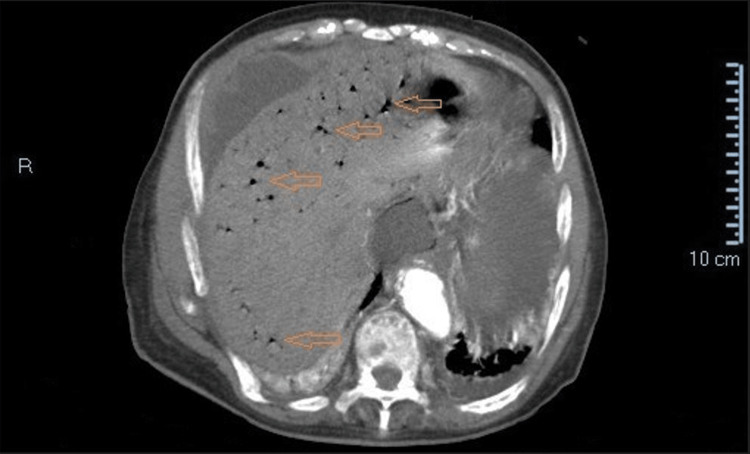
A contrast-enhanced CT scan shows exuberant signs of aeroportia (orange arrows)

We present the case of a 64-year-old hypertensive woman on regular hemodialysis due to chronic kidney disease of unknown etiology, with no other significant past medical history. There was no history of previous surgery or recent endoscopic examination. She was admitted to the hospital due to abdominal pain, vomiting, and diarrhea with a three-day course. At hospital admission, she was apyretic, prostrate, hypotensive, with mild abdominal pain. Initial laboratory tests revealed a slight increase in inflammatory parameters (leucocytosis of 13,000 uL, and C-reactive protein level of 8 mg/dL), with no other findings. She was started on intravenous fluid replacement, bowel rest, and antibiotic therapy. Contrast-enhanced CT was performed showing pneumatosis intestinalis suggestive of ischemic abnormalities, and aeroportia (Figures 1-2). No other major changes were found, and blood flow of the aorta, celiac trunk, and mesenteric arteries seemed to be preserved.

The patient was proposed for emergent exploratory laparotomy. However, she developed shock with rapidly worsening general condition and died within a few hours.

## Discussion

Aeroportia has rarely been described in chronic hemodialysis patients. To the best of our knowledge, only three cases have been described in the literature, and all of them presented with abdominal pain. Two cases reported no evidence of bowel necrosis or perforation, so aeroportia seemed to be caused by nonocclusive mesenteric ischemia (NOMI) and had a favorable outcome [5,6]. The third is a documented case of aeroportia and pneumatosis in the context of severe mesenteric atherosclerosis in a patient who was on chronic hemodialysis who died soon afterwards [7]. The pathogenesis is not fully understood, with three main mechanisms suggested: (1) mucosal disruption, with a breakdown of the endothelial barriers that may be seen in an ulcerative lesion, (2) presence of gas-forming bacteria that gain access to the portal venous system through the intestinal wall, and (3) increased intraluminal pressure where stretching enables gas to be absorbed rapidly [2,3,8]. Hemodialysis-dependent patients seem to be at risk of developing this condition. On the one hand, these patients harbor an altered gut microbial flora. During hemodialysis, the cell-to-cell structure may be compromised and favor bacterial translocation. On the other hand, prolonged hypotension with consequent mesenteric vasospasm of the peripheral arterioles leads to a decreased intestinal blood flow and may cause mucosal damage. This clinical entity, known as nonocclusive mesenteric ischemia, may allow the passage of intraluminal gas with no evidence of intestinal necrosis, and is an increasingly recognized complication in hemodialysis-dependent patients [6]. The association of aeroportia carries a worse prognosis and an increased mortality rate (75%-85%) [2,3,9,10]. Although the presence of aeroportia confers a worse prognosis, this is not dependent on its extent.

As mentioned earlier, mortality is high among patients with aeroportia and even higher if related to mesenteric ischemia. Our patient was a case of PI and aeroportia with hemodialysis dependence, presenting with abdominal pain, vomiting, and diarrhea with a three-day course. Although rarely observed, these radiological findings are characteristic of mesenteric ischemia. Our patient presented a rapid and progressive worsening of her clinical condition and she died soon afterwards. Although she never underwent an exploratory laparotomy, the contrast-enhanced CT scan showed no evidence of transmural necrosis or bowel perforation. In this case, since mesenteric ischemia was the main cause of intestinal pneumatosis and aeroportia, these findings seemed to be caused by NOMI, an increasingly recognized complication in hemodialysis patients. In addition, CT allowed the exclusion of other possibly associated causes, such as intra-abdominal abscesses, diverticulitis, and pancreatitis, among others. As a predictor of poor prognosis, the presence of aeroportia should be noted given the patient's unfavorable evolution and fatal outcome [3]. This case highlights the importance of early recognition of pneumatosis intestinalis and aeroportia. These entities are a rare but potential cause of life-threatening abdominal pain, especially in hemodialyzed patients who seem to be at risk of its occurrence [5].

## Conclusions

Hemodialysis-dependent patients should be considered at risk of the “low-flow syndrome” of mesenteric arterial circulation. However, it should not be overlooked that atherosclerotic disease with the involvement of the mesenteric vasculature is prevalent in this patient population. When pneumatosis intestinalis is present on CT, it is important to rule out potentially life-threatening complications, such as aeroportia that carries a worse prognosis and increased mortality. However, whether surgical intervention is indicated must be determined according to the clinical features of the individual patient. Early detection of these entities is crucial for the prompt diagnosis and management of mesenteric ischemia.
